# Major risk factors for *Streptococcus dysgalactiae* subsp. *equisimilis* bacteremia: a population-based study

**DOI:** 10.1186/s12879-023-07992-9

**Published:** 2023-01-23

**Authors:** Viivi Nevanlinna, Reetta Huttunen, Janne Aittoniemi, Tiina Luukkaala, Sari Rantala

**Affiliations:** 1 Department of Internal Medicine, Tampere University Hospital, Elämänaukio, Kuntokatu 2, 33520 Tampere, Finland; 2 Faculty of Medicine and Health Technology, Tampere University, Tampere, Finland; 3 Fimlab Laboratories, Tampere, Finland; 4 Research, Development and Innovation Center, Tampere University Hospital, Tampere, Finland; 5 Health Sciences, Faculty of Social Sciences, Tampere University, Tampere, Finland

**Keywords:** *Streptococcus dysgalactiae* subspecies *equisimilis*, Group C streptococci, Group G streptococci, Bacteremia, Risk factor, Obesity

## Abstract

**Background:**

*Streptococcus dysgalactiae* subspecies *equisimilis* is a human pathogen causing severe invasive infections. Detailed information on *S. dysgalactiae* subsp. *equisimilis* bacteremia and especially of predisposing factors are lacking. The purpose of the study is to investigate the risk factors of *S. dysgalactiae* subsp. *equisimilis* bacteremia compared to the general population in Finland.

**Methods:**

We retrospectively reviewed all patients older than 18 years with *S. dysgalactiae* subsp. *equisimilis* bacteremia in the Pirkanmaa health district from August 2015 to July 2018. The risk factors for *S. dysgalactiae* subsp. *equisimilis* bacteremia were investigated with respect to the normal population in Finland using the Finhealth study data provided by the Finnish institute for health and welfare. The study group was matched with the Finhealth study by age and sex.

**Results:**

Altogether 230 cases of S. *dysgalactiae* subsp. *equisimilis* bacteremia were detected. The medical records of 217 episodes of *S. dysgalactiae* subsp. *equisimilis* bacteremia (involving 211 patients) were available for analysis. Obesity was a statistically significant risk factor for *S. dysgalactiae* subsp. *equisimilis* bacteremia (Odds Ratio 2.96 [95% CI 2.22–3.96]). Diabetes and coronary artery disease were also associated with an increased risk of *S. dysgalactiae* subsp. *equisimilis* bacteremia (OR 4.82 [95% CI 3.62–6.42]) and (OR 3.03 [95% CI 2.18–4.19]).

**Conclusions:**

We found obesity, diabetes, and coronary artery disease to be associated with an increased risk for *S. dysgalactiae* subsp. *equisimilis* bacteremia. These results provide an increased understanding of risk factors for *S. dysgalactiae* subsp. *equisimilis* bacteremia.

## Background

Almost all ß-hemolytic, large colony-forming group C and G streptococci that infect humans form a single subspecies: *Streptococcus dysgalactiae* subspecies *equisimilis* [1]. The clinical spectrum of *S. dysgalactiae* subsp. *equisimilis* is similar to *Streptococcus pyogenes*, causing pharyngitis, skin and soft tissue infections, and severe invasive infections including endocarditis, septic arthritis, osteomyelitis, pneumonia, necrotizing fasciitis and Streptococcal toxic shock syndrome [2–4]. In several studies, the incidence of *S. dysgalactiae* subsp. *equisimilis* bacteremia has been increasing [4–7]. *S. dysgalactiae* subsp. *equisimilis* and *S. pyogenes* share virulence factors. There is some evidence that a few particularly virulent *S. dysgalactiae* subsp. *equisimilis* clones comprises increasing amount of the *S. dysgalactiae* subsp. *equisimilis* bacteremia cases. It is suggested that they could cause more invasive infections and the clinical picture might be more serious than earlier [8, 9].

Compared to *S. pyogenes*, *S. dysgalactiae* subsp. *equisimilis* bacteremia patients are known to be elderly individuals with many chronic diseases [10]. In case series, *S. dysgalactiae* subsp*. equisimilis* bacteremia patients are described to have several underlying conditions affecting their health, such as diabetes, cardiovascular disease, malignancy and alcohol use [3, 7, 10, 11]. Population based studies of S. *dysgalactiae* subsp. *equisimilis* bacteremia are limited [2–5, 12–15]. In one large American study, cardiovascular disease, diabetes, obesity and chronic skin disease were the most common underlying disease in *S. dysgalactiae* subsp. *equisimilis* bacteremia patients [2]. To our knowledge, there are no previous controlled studies on the risk factors for *S. dysgalactiae* subsp. *equisimilis* bacteremia.

In several studies, obesity and diabetes have been associated with an increased risk of several infections, including pneumonia, skin infections, and surgical site infections [16–18]. Age, male gender, diabetes, alcoholism, lung disease and cancer have been found to be risk factors for sepsis and bacteremia [12]. In a population-based study carried out in the United States, morbid obesity (BMI > 40) was associated with an increased risk of sepsis [19]. Langley et al. studied patients with invasive *S. pyogenes* infections and reported similar results: Morbid obesity was associated with a higher risk of sepsis and increased odds of death [20].

Detailed knowledge on risk factors for *S. dysgalactiae* subsp. *equisimilis* bacteremia is essential in order to target preventive measures and lower the risk for *S. dysgalactiae* subsp. *equisimilis* bacteremia. Our study presents the first population-based, controlled study of the major risk factors for *S. dysgalactiae* subsp. *equisimilis* bacteremia. The objective of the study was to compare the risk factors of *S. dysgalactiae* subsp. *equisimilis* bacteremia to risk factors in the general Finnish population.

## Methods

This population-based study was conducted in the Pirkanmaa health district, the second largest health district in western Finland with 535,044 inhabitants. The health district comprises one tertiary care hospital (Tampere University Hospital) and regional hospitals in Hatanpää, Valkeakoski, Vammala and Jämsä, and several smaller primary/secondary care units. All positive blood culture samples are registered in the Finnish register for hospital infections and antimicrobial use (SAI). All blood culture samples in the Pirkanmaa area are studied and cultivated in the Fimlab laboratories, Tampere. Blood cultures were collected in BacT/Alert Aerobic (FA Plus) and Anaerobic (FN Plus) blood culture bottles and incubated in an automated microbial detection system, BacT/Alert 3D (bioMérieux, Marcy l’Etoile, France), from August 2015 to October 2017, and in BD BACTEC Plus Aerobic/F and Lytic/10 Anaerobic/F culture vials and incubated in a BD BACTEC FX blood culture system (Becton Dickinson, Sparks, MD, USA) from November 2017 to July 2018. *S. dysgalactiae* subsp. *equisimilis* was primarily identified based on the large colony-forming typical growth and β-hemolysis on blood agar plates. Until 2017, the indentification was based on latex agglutination of the Lancefield group (PathoDxtraTM Strep Grouping Kit, Thermo Scientific, Basingstoke, Hants, UK) and API^®^ 20 STREP (bioMérieux, Marcy l’Etoile, France), which gave the specific identification to the species level. Since 2017, matrix-assisted laser-desorptionionization time-of-flight i.e., MALDI-TOF (VITEK^®^ MS, bioMérieux, Marcy l’Etoile, France) method was used for specific identification. MALDI-TOF gives the result in the form as *S. dysgalactiae* subsp. *dysgalaciae/equisimilis*, interpreted as *S. dysgalactiae* subsp. *equisimilis* associated with human disease.

We retrospectively identified patients over 18-years of age with one or more positive blood culture for *Streptococcus dysgalactiae* subsp. *equisimilis.* The study period was three years, from August 2015 to July 2018. During the study period, altogether 230 positive blood cultures were identified. Medical records were not available for 13 patients. The remaining 217 episodes of *S. dysgalactiae* subsp. *equisimilis* bacteremia (involving 211 patients) comprised the present study population. An infectious disease specialist (SR) reviewed all of the electronic patient records and filled in a structured case report form. The obesity status was determined by the patients’ body mass index (BMI) > 30. BMI was calculated by the standard formula (weight/heigt^2^). Diabetes included type 1 and 2 and was determined by HbA1c > 48 mmol or fP-gluk ≥ 7 mmol/l. Alcoholism was defined as a known social or medical problem due to alcohol, or alcohol usage exceeding 23 units or more per week for men and 12 units or more for women. Other chronic diseases were defined as according to the treating physician. Our study was approved by the Regional Ethics Committee of Tampere University Hospital.

### The Finhealth 2017 study

The underlying conditions of *S. dysgalactiae* subsp. *equisimilis* bacteremia patients were compared to the National Finhealth Study 2017 provided by the Finnish institute for health and welfare [21]. The study protocol is described in detail elsewhere [22]. Briefly, the Finhealth study is a population-based study on the health of the Finns. The study was carried out in 2017 with a study population of 10,000 randomly selected individuals over the age of 18. The study consists of questionnaires and a physical examination with measurements and blood sampling. The Finhealth study was approved by the Ethics Committee for the Helsinki-Uusimaa hospital district. The comparison between the study group and the Finhealth 2017 study was done for conditions available in the Finhealth study: obesity, diabetes, coronary artery disease, asthma, chronic obstructive pulmonary disease (COPD), and smoking. The study group was matched with the Finhealth study by sex and age groups (18–49 years, 50–64 years, 65–79 years and ≥ 80 years).

### Statistical methods

Statistical analyses were performed with the IBM SPSS Statistics for Mac, Version 27 (Armonk, NY: IBM Corp). The association of the risk factors for *S. dysgalactiae* subsp*. equisimilis* bacteremia between the study group and the Finhealth study were tested using the χ^2^ test or the Fisher exact test as appropriate. Study group was compared to the Finhealth study population using crosstabulations with Mantel–Haenszel Common Odds Ratio estimates. Odds ratios were expressed with 95 confidence intervals. P-values under 0.05 were considered statistically significant.

## Results

**Fig. 1:**
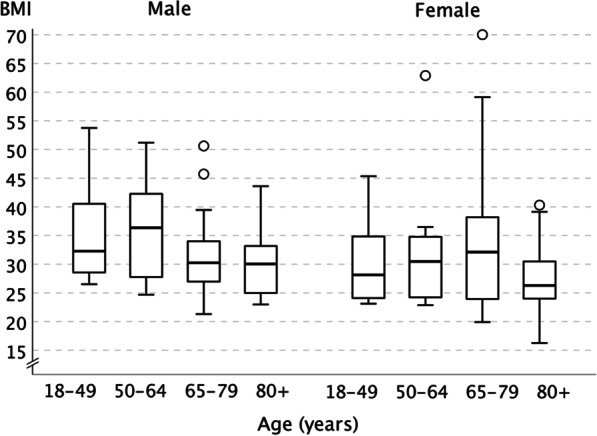
Distribution of BMI according to age and sex in Patients with *Streptococcus dysgalactiae* subsp. *equisimilis* bacteremia. Medians (black line), Interquartile ranges (box) and ranges (line bar) are given. Outliers are expressed as dots

During the 3-year study period, 230 cases of *S. dysgalactiae* subsp*. equisimilis* bacteremia were detected. The medical records of 217 episodes of *S. dysgalactiae* subsp*. equisimilis* bacteremia (involving 211 patients) were available for analysis. The clinical characteristics of *S. dysgalactiae* subsp*. equisimilis* patients are shown in Table 1. The majority of the patients were over 65 years old (77%), with a median age of 75 years (range 28–95 years). Sixty percent were male. The median BMI of the study patients was 30 (range 16–70). Forty four percent of the patients were significantly overweight (BMI > 30) and 9% (n = 20) morbidly obese (BMI > 40). The distribution of the BMI is shown in Fig. 1.

**Table 1:** Characteristics of Patients with *Streptococcus dysgalactiae* subsp*. equisimilis* bacteremia

Characteristic	Patients with *Streptococcus dysgalactiae* subsp. *equisimilis* bacteremia, N = 217	
Age years, n (%)		
18–49	17	(8)
50–64	33	(15)
≥ 65	167	(77)
65–79	83	(38)
≥ 80	84	(39)
Age, Median (IQR)	75	(65–83)
Male, n (%)	130	(60)
BMI, n (%)		
< 25	43	(20)
25.0–30.0	50	(23)
> 30	95	(44)
Missing	29	
BMI, Median (IQR)	30	(25–36)
Underlying conditions, n (%)		
Current smoker	24	(11)
Alcohol abuse	27	(12)
Skin conditions	138	(63)
Diabetes	81	(37)
Heart disease	97	(45)
* Coronary artery disease*	53	(24)
* Cardiac insufficiency*	74	(34)
Chronic pulmonary disease	37	(17)
* COPD*	14	(7)
* Asthma*	20	(9)
Chronic kidney disease	30	(14)
Chronic liver disease	10	(5)
Neurological disease	44	(20)
Malignancy	62	(29)
Immunosuppression	28	(13)
No underlying disease	3	(1)

n, number of *Streptococcus dysgalactiae* subsp*. equisimilis* bacteremia patients; IQR interquartile range, BMI body mass index, COPD, chronic obstructive pulmonary disease

Almost all patients (214 of 217) had one or more underlying condition affecting their health. The most common underlying chronic diseases were heart diseases (45%) including coronary artery disease and cardiac insufficiency, and diabetes (37%). Several patients had more than one underlying conditions; 25% was obese and had diabetes and 6% had obesity, diabetes, and coronary artery disease. Twentynine percent of the patients had malignancies, the most common malignancies being breast cancer (33%), gynecological cancer (10%), hematological cancer (11%) and prostate cancer (11%). Other chronic diseases are shown in Table 1. Skin conditions were the most common underlying health problem (63%). Fifty-eight percent of all patients had chronic eczema or skin erosion. Twenty percent had a chronic ulcer and 4% a traumatic wound. Seventeen percent of patients had a preceding trauma during the previous month and 2 patients (1%) had a surgical wound. Some patients had more than one type of erosion. There were no patients with an HIV infection and no patients were known users of intravenous drugs.

Risk factors for *S. dysgalactiae* subsp*. equisimilis* bacteremia in the study group compared to the controls in the Finhealth study are shown in Table 2. Obesity was a statistically significant risk factor for *S. dysgalactiae* subsp*. equisimilis* bacteremia (OR 2.96 [95% CI 2.22–3.96], p < 0.001). Obesity increases the risk of *S. dysgalactiae* subsp*. equisimilis* bacteremia both in males and females (Table 2). Compared to the Finhealth controls, the portion of obese males both in age groups under 50 years and over 80 years were significantly higher in the study group, and the risk of *S. dysgalactiae* subsp*. equisimilis* bacteremia seems to be remarkably increased both in young and aged obese males (Table 2).

**Table 2:** Risk factors for *Streptococcus dysgalactiae* subs*. equisimilis* bacteremia according to the age groups in the study group compared to controls in the Finhealth 2017 study

Age years	Male									Female								
	Cases			Controls			Cases vs. controls			Cases			Controls			Cases vs. controls		
	N	n	(%)	N	n	(%)	P-value	OR	(95% CI)	N	n	(%)	N	n	(%)	P-value	OR	(95% CI)
**Obesity**	**117**	**66**	**(56)**	**2701**	**685**	**(25)**	** < 0.001**	**3.81**	**(2.62–5.55)**	**74**	**32**	**(43)**	**3158**	**853**	**(27)**	** < 0.001**	**2.06**	**(1.29–3.28)**
18–49	8	5	(63)	1108	241	(22)	0.016	6.00	(1.42–25.27)	8	3	(38)	1244	256	(21)	0.374	2.32	(0.55–9.75)
50–64	18	12	(67)	833	260	(31)	0.003	4.41	(1.64–11.87)	11	6	(55)	925	282	(30)	0.103	2.74	(0.83–9.04)
65–79	56	30	(54)	656	167	(26)	< 0.001	3.38	(1.94–5.88)	24	14	(58)	810	252	(31)	0.007	3.10	(1.36–7.07)
≥ 80	35	19	(54)	139	36	(16)	< 0.001	6.08	(2.61–14.13)	31	9	(29)	179	63	(35)	0.546	0.75	(0.33–1.73)
**Diabetes**	**130**	**54**	**(42)**	**2694**	**360**	**(13)**	** < 0.001**	**4.61**	**(3.19–6.64)**	**87**	**27**	**(31)**	**3169**	**285**	**(9)**	** < 0.001**	**4.55**	**(2.85–7.29)**
18–49	9	1	(11)	1094	34	(3)	0.253	3.90	(0.47–32.04)	8	1	(13)	1254	32	(3)	0.191	5.46	(0.65–45.65)
50–64	20	9	(45)	831	131	(16)	0.002	4.37	(1.78–10.76)	13	4	(31)	921	76	(8)	0.020	4.94	(1.49–16.42)
65–79	58	29	(50)	661	171	(26)	< 0.001	2.87	(1.66–4.93)	25	10	(40)	811	143	(18)	0.014	3.11	(1.37–7.07)
≥ 80	43	15	(35)	108	24	(22)	0.148	1.88	(0.87–4.07)	41	12	(29)	183	34	(19)	0.137	1.81	(0.84–3.93)
**Coronary artery disease**	**130**	**35**	**(27)**	**1936**	**230**	**(12)**	** < 0.001**	**2.73**	**(1.81–4.12)**	**87**	**18**	**(21)**	**2301**	**179**	**(8)**	** < 0.001**	**3.09**	**(1.80–5.31)**
18–49	9	2	(22)	61	0	(0)	0.015	–		8	0	(0)	81	1	(1)	1.000	–	
50–64	20	0	(0)	956	64	(7)	0.636	–		13	0	(0)	1025	30	(3)	1.000	–	
65–79	58	15	(26)	775	126	(16)	0.069	1.80	(0.97–3.33)	25	4	(16)	940	81	(9)	0.268	2.02	(0.67–6.03)
≥ 80	43	18	(42)	144	40	(28)	0.092	1.87	(0.92–3.80)	41	14	(34)	255	67	(26)	0.345	1.46	(0.72–2.94)

N, total number of cases and contols; n, number of cases and controls with obesity, diabetes and coronay artery disease

Diabetes was a risk factor for *S. dysgalactiae* subsp*. equisimilis* bacteremia (OR 4.82 [95% CI 3.62–6.42], p < 0.001). The risk was increased both in males and females, being higher in the age groups under 65 years, than in the older age groups (Table 2). Coronary artery disease was also a risk factor for *S. dysgalactiae* subsp*. equisimilis* bacteremia (OR 3.03 [95% CI 2.18–4.19], p < 0.001). The risk was statistically significant in both males and females. In the subgroup analyses according to age the risk did not reach statistical significance.

The risk factors were studied in relation to age groups. As shown in Table 2, obesity was most common in age group 50–64 years (62%) and least common in the oldest age group (42%), (p = 0.275). Diabetes peaked in patients aged 50–64 (45%) and 65–79 (59%), (p = 0.028). The proportion of patients with coronary artery disease was highest in the oldest age group (38%), (p < 0.001). In patients with one or more of the three risk factors (obesity, diabetes, and coronary artery disease), the distribution between the age groups were even (72–80%), (p = 0.815). In patients with 2/3 risk factors, most patients were aged 50–64 and 65–70 years (35% each), (p = 0.021). In patients with all three risk factors, the distribution was as follows: 18–49 years (0%), 50–64 years (0%), 65–79 years (8%), and ≥ 80 years (7%), (p = 0.294).

We also studied the role of asthma (OR 0.72 [95% CI 0.45–1.15], p 0.21), COPD (OR 1.54 [95% CI 0.87–2.72], p 0.19) and smoking (OR 1.75 [95% CI 0.76–1.82], p 0.48) as risk factors for *S. dysgalactiae* subsp*. equisimilis* bacteremia, but no statistically significant association was found in the full study group nor in the subgroup analyses. Other underlying conditions, including malignancy, immunosuppression, skin conditions, neurological disease and alcohol use were not compared due to missing or incomparable data in the Finhealth 2017 study.

## Discussion

In this population-based controlled study, obesity, diabetes, and coronary artery disease were found to be associated with an increased risk for for *S. dysgalactiae* subsp*. equisimilis* bacteremia. This is the first study presenting obesity and coronary artery disease as risk factors for *S. dysgalactiae* subsp*. equisimilis* bacteremia. There is one previous study about the association of diabetes and an increased risk for *S. dysgalactiae* subsp*. equisimilis* bacteremia [23].

There are several studies concerning the risk of infections in obese patients. An association with obesity and an increased risk of infections have been previously reported in skin and soft tissue infections and surgical site infections [17, 24]. Kaspersen et al. studied hospital-treated infections and found that male patients had a higher incidence of skin and soft tissue infections and that the risk of skin and soft tissue infections was increased in obese males [16]. Morbid obesity has been reported to be associated with an increased risk for sepsis [19]. In an American study on invasive *Streptococcus pyogenes* infections, morbid obesity was also associated with an increased risk for invasive *S. pyogenes* infections [20]. In one non-controlled population-based study of *S. dysgalactiae* subsp. *equisimilis* bacteremia by Broyles et al., 30% of the patients were reported obese [2].

Several mechanisms for how obesity predisposes to infections have been suggested. Obesity weakens the skin barrier and wound healing and decreases respiratory capacity. Adipose tissue participates in the immune system producing several proinflammatory and anti-inflammatory factors called adipokines. Adipokines have several complex effects on different immune cells, mediating cytokine production in immune cells, cell differentiation and proliferation. [25, 26] Likewise in several other European countries and America, the prevalence of obesity has increased dramatically in Finland over the last two decades. In 1997, 15% of the population were obese, and in 2017 the prevalence of obesity had increased to 23% [21, 27].

In our study, diabetes increases the risk of *S. dysgalactiae* subsp. *equisimilis* bacteremia 5-times in the full study group. Diabetes has previously been associated with many severe infections including pneumonia, skin and subcutaneous infections, and sepsis [18, 28]. Thomsen et al. studied the role of diabetes in *S. pyogenes*, *S. agalactiae*, and *S. dysgalactiae* subsp. *equisimilis* bacteremia and found diabetes to be risk factor for bacteremia in *S. agalactiae* and *S. dysgalactiae* subsp. *equisimilis* [23]. In other studies, diabetes has also been associated with increased risk for *S. pyogenes* bacteremia [20, 29]. In previous case series, 16 to 42% of the patients with *S. dysgalactiae* subsp. *equisimilis* bacteremia have had diabetes [2, 3, 7, 14]. Ogura et al. studied the pathogenicity of *S. dysgalactiae* subsp. *equisimilis* with diabetic mice and found that the pathogenicity of *S. dysgalactiae* subsp. *equisimilis* was higher in T2DM model mice than in nondiabetic mice [30].

Coronary artery disease was also found to be a risk factor for *S. dysgalactiae* subsp. *equisimilis* bacteremia both in males and in females. In previous studies, some evidence for cardiovascular diseases increasing the risk of invasive *S. pyogenes* infections and other septic infections has been reported [29, 31].

The prevalence of other underlying conditions, including alcohol use, immunosuppression, skin conditions and malignancies, and are in line with previous studies [2, 3, 7, 14].

In the present study, the majority of *S. dysgalactiae* subsp. *equisimilis* bacteremia patients were elderly individuals, as in previous studies [2, 7, 14]. The reason for *S. dysgalactiae* subsp. *equisimilis* to affect mainly elderly individuals is multifactorial. Ageing predisposes to infections due to a higher prevalence of comorbidities. However, in this study, there were variation in the distributions of different risk factors according to age. The proportion of obese patients was larger in younger patients, diabetes was more common in the middlest age groups and coronary artery disease was emphasized in the oldest patients. Elderly individuals may have several comorbidities, but ageing is also known to increase the risk for infections by weakening the immune system [32]. Furthermore, the epidemiological features and changes concerning age may also be due to the bacteria itself. There is some evidence on horizontal gene transfer from *S. pyogenes* to *S. dysgalactiae* subsp. *equisimilis* that might affect the virulence of *S. dysgalactiae* subsp. *equisimilis* [33]*.* It seems that a few particularly virulent *S. dysgalactiae* subsp. *equisimilis* clone comprises increasing amount of the *S. dysgalactiae* subsp. *equisimilis* bacteremia cases, causing more invasive disease than earlier [8, 9, 34]. For example in Japan, where *S. dysgalactiae* subsp. *equisimilis* bacteremia is known to occur frequently, the emm stG6792 has been the predominant type [34]. In one previous study from Finland from 2010, the three most common emm-types were stG480, stG6 and stG485, comprising 51% of isolates, but more recent data is not available [15]. The genotype stG6792 was not reported in Finland at the time.

Our study has some limitations. We were not able to compare all possible risk factors for *S. dysgalactiae* subsp. *equisimilis* bacteremia due to the different methods in collecting data in the Finhealth study compared to the *S. dysgalactiae* subsp. *equisimilis* study group. Use of alcohol was determined differently in the Finhealth study, so the data was not comparable with our study group. Data of malignancies, immunosuppression and neurological diseases were not available in the Finhealth study.

Our study has several strengths. Both our study population and the Finhealth study group are population-based and both studies were carried out at the same time. Our study population is from Pirkanmaa health district and the Finhealth study represents the whole Finland. When different regions were compared in the Finhealth study, no significant regional differences were found [21]. In our study population, all records of patients were thoroughly reviewed by the same infectious disease specialist (SR). The BMI data was comprehensively available (87% of the patients).

## Conclusion

In conclusion, obesity, diabetes, and coronary artery disease were associated with an increased risk for *S. dysgalactiae* subsp. *equisimilis* bacteremia. Our results provide an increased understanding of risk factors for *S. dysgalactiae* subsp. *equisimilis* bacteremia.

## Data Availability

The dataset is not publicly available due to individual privacy but are available from the corresponding author on reasonable request.
